# Retraction: Quantitative GABA magnetic resonance spectroscopy as a measure of motor learning function in the motor cortex after subarachnoid hemorrhage

**DOI:** 10.3389/fneur.2026.1971327

**Published:** 2026-08-21

**Authors:** Frontiers Editorial Office

The authors retract the article cited above.

Following publication, the authors contacted the Editorial Office to request the retraction of the cited article, stating that serious problems were identified. Errors were found in the age, WFNS grade, and GABA measurement values for the coiling cases in Table 1. The data are outdated and cannot be corrected, as the MRI scanner that was used for the data collection is no longer available. The conclusions reported in the article are no longer supported by the data. An investigation was conducted in accordance with Frontiers' policies that confirmed this; therefore, the article has been retracted.

This retraction was approved by the Chief Editor of *Frontiers in Neurology - Neurological Biomarkers* and the Chief Executive Editor of Frontiers. The authors agree to this retraction.

